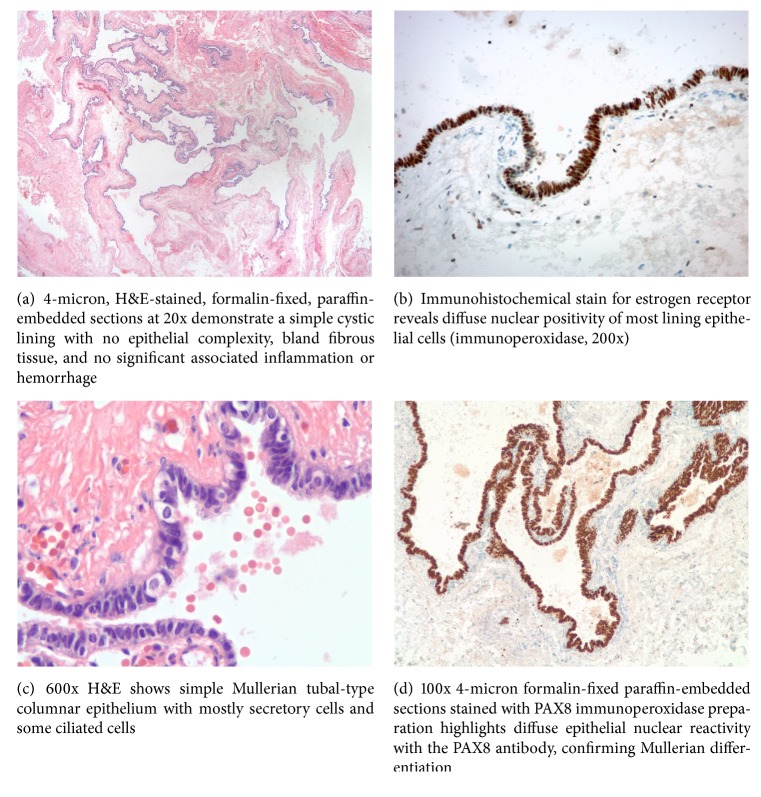# Robot-Assisted Thoracoscopic Resection of a Posterior Mediastinal Mullerian Cyst

**DOI:** 10.1155/2018/1424275

**Published:** 2018-02-04

**Authors:** Calvin Chao, Vijay Vanguri, Karl Uy

**Affiliations:** ^1^Division of Thoracic Surgery, UMass Memorial Medical Center, University of Massachusetts Medical School, Worcester, MA, USA; ^2^Department of Pathology, UMass Memorial Medical Center, University of Massachusetts Medical School, Worcester, MA, USA

## Abstract

First described in 2005, the Mullerian derived cyst in the mediastinum is a rare finding with few subsequent reports. We report a case of Mullerian cyst occurring in the mediastinum of a 49-year-old female that was resected by robot-assisted thoracoscopic surgery. To our knowledge, this is the first report of robot-assisted resection of Hattori's cyst. Histopathologic analysis revealed ciliated Mullerian-type tubal epithelium positive for paired box gene 8 (PAX8), estrogen receptor (ER), and progesterone receptor (PR), confirming Mullerian differentiation. We also review the clinical presentation, pathology, and differential diagnosis of such cysts.

## 1. Introduction

The Mullerian mediastinal cyst was first described by Hattori in 2005 [1]. He reported a posterior mediastinal cyst in an 18-year-old woman with no anatomic abnormalities or clinical symptoms. Since then, a number of additional cases have been reported [2]. Moreover, it appears that Mullerian mediastinal cysts are more common than once thought as retrospective analyses reveal a higher than expected percentage of mediastinal cysts which are Mullerian in origin [3]. We report a case of Hattori's cyst occurring in the posterior mediastinum of a 49-year-old female patient that was resected by robot-assisted thoracoscopic surgery (RATS).

## 2. Case Report

**Figure 1:**
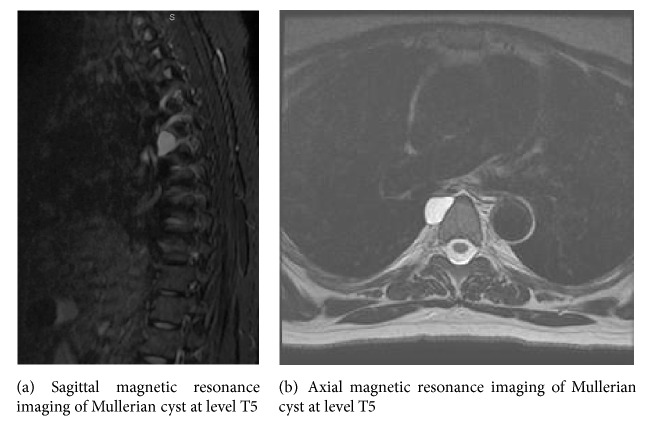


A 49-year-old woman presented to our clinic for the first time in consultation because of an incidental finding of a paraspinal mediastinal cyst. She was a healthy-appearing, obese (BMI 30.6) woman who recently moved from West Virginia. She is gravida 1, para 1, with first child born when she was 18 years old. She currently has an intrauterine device and previously took oral contraceptive pills for 15 years. The patient was status post-C5-C6 anterior cervical discectomy and fusion for spinal compression. She has a strong family history of breast cancer and is Necdin variant positive (NDN). This gene, located in the Prader-Willi syndrome deletion region, facilitates cell cycle arrest, predisposing her to development of breast cancer. The patient has denied prophylactic mastectomy. During workup for migraine headaches and cervicalgia, a magnetic resonance imaging (MRI) scan of her cervical spine was performed, leading to an MRI of the thoracic spine. In this subsequent MRI, a well-circumscribed homogeneous mass with signals compatible with a pure cyst measuring 2.0 × 1.2 × 2.2 cm was found along the right anterolateral aspect of the T5 vertebral body (Figure 1).

Though we recommended continued monitoring of the cyst, the patient, given her genetic predisposition and family history of cancer, opted for surgical intervention. The patient underwent robot-assisted thoracoscopic surgery for resection of the cyst for diagnostic and therapeutic purposes.

After induction of general anesthesia utilizing a double-lumen endotracheal tube, the patient was placed on the left lateral decubitus position and the da Vinci Si robot was docked. We utilized 4 ports placed along the 7th intercostal space with CO2 insufflation and a 0-degree 10 mm camera. After retracting the lung anteriorly, we located the cyst at the paraspinal T5 level, where it was encompassed by veins draining to the azygos system. Though the cyst was mostly adherent to the body of the spine, we were able to find a distinct plane for complete excision. The cyst wall was inadvertently ruptured during the dissection and drained serous fluid but was excised completely. The patient had a severe migraine postoperatively but otherwise had a good recovery and was discharged on the second postoperative day.

Gross pathological examination revealed the paraspinal cyst to be a 1.3 × 1.0 × 0.3 cm previously disrupted pink-red cystic structure which was bisected to reveal a smooth inner and outer lining. Histopathologic analysis revealed a cyst lined by ciliated Mullerian-type tubal epithelium with no evidence of malignancy. Immunohistochemical staining with paired box gene 8 (PAX8), estrogen receptor (ER), and progesterone receptor (PR) was done to confirm a Mullerian origin as was also described by Hattori [4] (Figure 2).

## 3. Discussion

To our knowledge, this is the 19th documented case in the English literature of a Mullerian cyst in the mediastinum and the first reported case of such a cyst resected by robot-assisted thoracoscopic surgery [2]. Of the three mediastinal compartments, the posterior mediastinum is the least likely to contain malignant masses and is amenable for thoracoscopic approach. Important anatomic considerations in the posterior mediastinum include the descending thoracic aorta, trachea, esophagus, azygous venous system, and potential spinal involvement. Given the paraspinal location, low likelihood of malignancy, and proximity to important structures, a robotic approach was preferred. The precise manipulation of the machine arms enabled complete excision of the cyst from the spine without damage to the encompassing veins.

We considered other differential diagnoses in assessing this posterior mediastinal mass. Neoplasms such as schwannomas, neuroblastomas, ganglioneuroblastoma, gangliomas, and spinal cord lesions often appear in the posterior mediastinum but are not cystic and can be ruled out preoperatively by imaging and lack of clinical manifestation. An anterior spinal meningocele may also present in the posterior mediastinum and will appear cyst-like on imaging due to filling of cerebrospinal fluid. However, the meningocele will communicate with the vertebral column unlike a true cyst.

The most common mediastinal cysts include bronchogenic cysts, enteric duplication cysts, pericardial cysts, and thymic cysts, though most of these cysts appear in the middle mediastinum with the exception of thymic cysts in the anterior mediastinum. Bronchogenic cysts can be ciliated and may stain positive cytokeratin (CK7) positive but rarely stain PAX8, WT-1, and ER positive [5]. Enteric duplication cysts may be ciliated and have been reported to stain PAX8 positive or CK7 positive but rarely stain PR and ER positive [6]. Pericardial cysts are quite rare (1/100,000) and typically present in the right cardiophrenic angle. They may stain cytokeratin positive but again rarely stain PAX8, ER, and PR positive. Thymic cysts are similarly quite rare and are typically a single cyst associated with a soft tissue component and connection to thymic tissue. Mesothelial cysts are another consideration but are typically sequelae of previous surgery. Therefore, having ruled out the likelihood of the above presentations, we may reasonably conclude that our cyst, having stained for CK7, WT-1, PAX8, ER, and PR, is Hattori's cyst.

The pathogenesis of a posterior mediastinal Mullerian cyst is still not yet completely understood. Previous reported cases of Hattori's cysts have posited that such cysts are the result of remnant Mullerian tissue during embryogenesis as proposed by Ludwig et al. in his study of Mayer-Rokitansky-Kuster syndrome [7, 8]. The first Mullerian duct anlage arises in the 3rd to 5th thoracic vertebral blastema in stage 16 embryos. At stage 17, the Mullerian duct appears via a deep invagination at the level of the 3rd thoracic somite and proceeds to grow caudally towards the Wolffian ducts until fusion in stage 18. The two Mullerian ducts will continue caudally until reaching the dorsal wall of the urogenital sinus at stage 23, forming the cervicovaginal canal. The cervicovaginal canal and nearby anlages will later form the location between the fallopian tube and uterine horn [7]. An aberration of this process is the root cause of Mullerian agenesis and remnant structures during this migration are perhaps also the pathogenesis of Hattori's cysts [9].

In conclusion, we present a patient with a posterior mediastinal mass identified to be a cyst of Mullerian origin. At her one-month postoperative clinic visit she denied any postoperative complications and endorsed only continued migraines and cervicalgia for which she will follow with her neurologist. While rare in presentation we review the diagnosis of Hattori's cysts and present the first documented RATS approach for resection with considerations for such a robotic approach. Lastly, we overview the likely pathogenesis of such cysts as a remnant of Mullerian duct migration.

## Figures and Tables

**Figure 2:**